# Health-related quality of life measures in chronic pulmonary aspergillosis: a systematic review of psychometric validity

**DOI:** 10.1183/16000617.0039-2026

**Published:** 2026-11-04

**Authors:** Dareen Marghlani, Aran Singanayagam, Zhifang Melody Ni, Darius Armstrong-James, Anand Shah

**Affiliations:** 1 Centre for Bacterial Resistance Biology, Department of Infectious Disease, Imperial College London, London, UK; 2 Imperial Fungal Science Network, Department of Infectious Disease, Imperial College London, London, UK; 3 Royal Brompton and Harefield Hospitals, Guy's and St. Thomas’ NHS Foundation Trust, London, UK; 4 London In-Vitro Diagnostic Co-operative, Department of Surgery and Cancer, Imperial College London, London, UK

## Abstract

**Background/Objective:**

Chronic pulmonary aspergillosis (CPA) is a chronic progressive fungal lung infection associated with global mortality and impact on health-related quality of life (HRQoL). HRQoL tools are valuable in disease management and as trial end-points; however, use in CPA has not been systematically analysed. We analyse CPA-specific symptoms and strengths and limitations of currently used HRQoL tools focusing on psychometric validity.

**Methods:**

A systematic review of studies reporting HRQoL tools or symptom scores in CPA and published in English (inception to May 2025) was conducted. Eligible studies were categorised into two groups, those employing HRQoL questionnaires (questionnaire-based studies (QBS) and those reporting CPA symptoms to analyse content validity. Narrative synthesis followed Synthesis Without Meta-analysis (SWiM) reporting guidance.

**Results:**

Of 520 studies identified, 64 met inclusion criteria, including 18 QBS. Only generic tools have measured HRQoL in CPA, with St. George's Respiratory Questionnaire (SGRQ) the most common. SGRQ demonstrated excellent internal consistency (Cronbach's alpha 0.94), test–retest reliability (intraclass correlation coefficient 0.97) and an anchor-based minimal clinically important difference of 7.3 points; an >8-unit change in the SGRQ symptoms domain discriminated clinical recurrence at 12 months with 90.9% sensitivity and 87.0% specificity. Significant limitations however remain regarding content validity and usability. Other HRQoL tools or symptom scores used had minimal psychometric validity.

**Conclusion:**

Current CPA HRQoL tools are generic with SGRQ the only tool with psychometric validity but limitations in content validity and usability highlighting the need for development of a CPA-specific HRQoL tool to aid clinical monitoring and standardisation of therapeutic end-points.

## Shareable abstract

**Chronic pulmonary aspergillosis (CPA) profoundly affects health-related quality of life (HRQoL); however, existing monitoring tools lack disease specificity, are complex or have limited validity, highlighting the need for a CPA-specific HRQoL tool.** https://bit.ly/4uFPBLQ

## Introduction

Chronic pulmonary aspergillosis (CPA) is a progressive pulmonary fungal infection caused by *Aspergillus* species, most commonly *Aspergillus fumigatus* [1]. Its prevalence has been estimated at ∼3 million people globally, with previous studies suggesting a ∼30% 5-year mortality [2]. CPA most commonly affects immunocompetent individuals or those with only mild underlying immunocompromise, often with structural underlying lung disease, with prior tuberculosis the most common predisposing condition globally. Other conditions predisposing to CPA are nontuberculous mycobacterial infection, allergic bronchopulmonary aspergillosis (ABPA), sarcoidosis and previous lung cancer, with COPD being the most common predisposing condition in the developed world [3–5]. Presentation is often insidious, with disease development often occurring over months or years, with diagnosis based on a constellation of clinical, radiological, microbiology and serological findings. Although there is a broad spectrum of CPA with radiological heterogeneity and variable symptoms with a range of severity depending on the underlying condition, there is often a deleterious consequence on health-related quality of life (HRQoL) [6].

HRQoL is a multidimensional approach that measures the overall well-being of individuals, focusing on the effect of a condition and the treatment impact on the individual's physical, mental, psychological and social well-being [7]. Measuring HRQoL helps assess the effectiveness of clinical interventions and treatments, offering insights into patients’ perceived health and well-being beyond clinical outcomes. Various HRQoL tools (also known as instruments or questionnaires) have been developed and used in different chronic conditions that involve robust development and validation phases, often based on input from patients, clinicians and experts to capture relevant aspects of HRQoL, such as symptom domains [8, 9]. HRQoL tool effectiveness is assessed through psychometric validation processes, including usability and acceptability. This includes testing for reliability (consistent results across time and populations), validity (accurately measuring the intended health concepts from the perspective of experts, including healthcare professionals and patients) and sensitivity (detecting meaningful changes in a patient's health status). Without demonstrated psychometric validity, the tool's results may be less trustworthy or applicable in clinical or research settings [10].

HRQoL tools are often classified as generic or disease-specific. Generic tools offer broad, cross-condition comparisons, where the overall impact of various diseases on populations is evaluated. However, they may lack sensitivity to disease-specific symptoms or changes in a patient's condition. Disease-specific tools, on the other hand, provide nuanced insights into the physical, emotional and social impact of a given condition, capturing key symptoms and treatment side-effects that may be overlooked in generic assessment [11]. Hence, disease-specific HRQoL scores have been widely developed and used to assess chronic pulmonary conditions such as COPD and asthma. They are regarded as important markers of disease progression and therapeutic efficacy and are embedded as gold-standard tools in different aspects of clinical practice and research. An example is the COPD Assessment Test (CAT), which quantifies the impact of COPD severity and symptoms on patients’ general health, is associated with disease severity and provides a reliable method to assess therapeutic response and prognosis [12, 13].

Although CPA was first known as a fatal disease in 1842 with known effects on HRQoL, to date, defining treatment outcomes in CPA has been challenging in part due to a lack of disease-specific HRQoL tools to measure the impact of the condition on the patient's health [14–16]. Similar to other chronic conditions, generic tools may not capture all symptom domains in CPA due to disease heterogeneity, residual symptoms from underlying lung conditions and limited sensitivity [16, 17]. As an example, prior studies highlighted problems using the Modified Medical Research Council scale (mMRC) and Baseline Dyspnea Index (BDI) as standalone scores to measure dyspnoea in COPD patients, as they may evaluate symptom severity very differently, resulting in the development of a novel self-administered disease-specific tool [18].

At present, there is a paucity of therapeutic clinical trial evidence in chronic pulmonary aspergillosis and a need for refined outcome definitions, including HRQoL, which is essential for evaluating and comparing the effectiveness of different therapeutic approaches [19]. An ideal tool for assessing HRQoL in chronic conditions should be valid, reliable and responsive, capturing the specific impacts of the disease on patients’ health [9]. It must also be simple, quick to complete and easy to score, ensuring practicality in routine clinical settings while providing meaningful data to guide patient management [20].

In this systematic review, we analyse how HRQoL is measured and assessed in patients with CPA. We analyse currently used HRQoL tools and symptom scores in CPA within a systematic framework to evaluate strengths and limitations with a focus on analysing the psychometric properties of these tools.

## Methods

### Data source and search strategies

Embase and Medline (Ovid) databases were searched from database inception to May 2025 to identify relevant publications that report HRQoL data in CPA. Search strategy (supplementary table S1) were developed using medical subject headings (MeSH) and text words related to the following concepts: chronic pulmonary aspergillosis (CPA and health-related quality of life (HRQoL): (“Chronic pulmonary aspergillosis” OR “CPA” OR “Aspergillus nodule” OR “simple aspergilloma” OR “chronic cavitary pulmonary aspergillosis” OR “CCPA” OR “chronic fibrosing pulmonary aspergillosis” or “CFPA”) AND (“Quality of Life” OR “QoL” OR “Health-Related Quality of Life” OR “HRQoL” OR “outcome measure” OR “outcome assessment” OR “symptom score” OR “symptoms scale” OR “healthcare surveys” OR “patient-reported outcome measures” OR “PROMs”). Additionally, reference lists were manually searched to identify any additional relevant articles.

### Eligibility criteria

To evaluate how HRQoL is currently measured and assessed in patients with CPA, the following inclusion criteria were applied to identify eligible studies: 1) published in the English language; 2) designed as observational studies (cohort, case–control, cross-sectional studies), experimental studies (clinical trials) and original, peer-reviewed articles; 3) reporting information on HRQoL in CPA either by using subjective or objective approaches to assess HRQoL/quality of life (QoL) outcomes. Irrelevant studies excluded included conference abstracts, book chapters, editorials, commentary, case reports, case series, letters, preprints, study protocols and those not written in English. Studies published reporting data on other forms of aspergillosis were also excluded.

### Data screening and selection process

Upon completing the search process, database search results were imported into Mendeley Reference Manager (www.mendeley.com) to track references. All titles and abstract references were transferred to Covidence, an electronic software for managing and streamlining the systematic review process for screening and data extraction (Veritas Health Innovation, Melbourne, Australia; www.covidence.org). Duplicate articles were removed and manually checked. Two independent reviewers (D. Marghlani and A. Shah) screened relevant titles, abstracts and full texts against eligibility criteria, with any disagreements settled by consensus. For each selected manuscript, the author;s name, year of publication, country of study, sample size, study design, information on HRQoL (symptom scores and HRQoL/QoL measures) and outcomes were reported on a predesigned data extraction sheet on Microsoft Excel.

### Outcomes

Primary outcomes were the strengths and limitations of current HRQoL tools and symptom scores used in CPA analysing psychometric properties, including content validity, internal consistency, test–retest reliability, sensitivity/responsiveness, minimal clinically important difference (MCID) and convergent validity. Secondary outcomes were usability and feasibility (time of administration, complexity, length and clarity). We additionally report as an outcome the CPA-specific symptom burden identified across nonquestionnaire-based studies (non-QBS).

### Data synthesis

All studies that met the inclusion criteria, regardless of study quality, were included for data synthesis (supplementary tables S2 and S3). A structured narrative synthesis was conducted following Synthesis Without Meta-analysis (SWiM) reporting guidance [21] (supplementary table S4). Studies were grouped for synthesis according to the protocol-specified strategy, as follows: 1) those employing HRQoL questionnaires or symptom score indices; or 2) those using objective clinical, immunological, radiological or microbiological assessment methods corresponding to our questionnaire-based studies (QBS) and non-QBS classification below. Meta-analysis was not performed owing to substantial heterogeneity in instruments, populations and outcome definitions across studies, consistent with the protocol. For each study, the methodological approaches adopted to assess HRQoL dimensions and validity among CPA patients were identified. An additional consensus study in CPA deemed relevant was included given the importance within the field [19]. A narrative synthesis of the findings was conducted to present the results and discuss the study. HRQoL findings, including subjective and objective approaches reported on CPA, were descriptively summarised. No risk of bias (quality) assessment was performed, as our purpose was to extract all relevant data on HRQoL use and validity without comparison [22].

### Articles classification strategy

Identified studies adopted different approaches and assessment methods. Those were classified into two main categories, namely QBS) and non-QBS. QBS included studies that utilised existing HRQoL tools and symptom scores. Non-QBS highlighted key clinical symptoms identified as relevant in CPA to report HRQoL outcomes and evaluated the effect or impact on clinical, immunological, radiological and microbiological outcomes. These studies did not specifically utilise HRQoL tools in their study designs but identified key clinical symptoms relevant to CPA, which were used to evaluate the psychometric properties, especially content validity, of existing HRQoL tools.

## Results

### Study selection and description

In total, 64 studies were selected and included in the review (figure 1). Of these, 18 QBS evaluating HRQoL in CPA were identified and 46 were non-QBS as defined above (supplementary tables S2 and S3). Among the 18 QBS, a global perspective was represented, most cohorts were from the UK (n=10) and India (n=5), with additional cohorts from France, Uganda and Ghana (n=1 each). Across the QBS, sample sizes ranged from 18 to 387 individuals with CPA, with a median of 91 (interquartile range 49–162).

**FIGURE 1:**
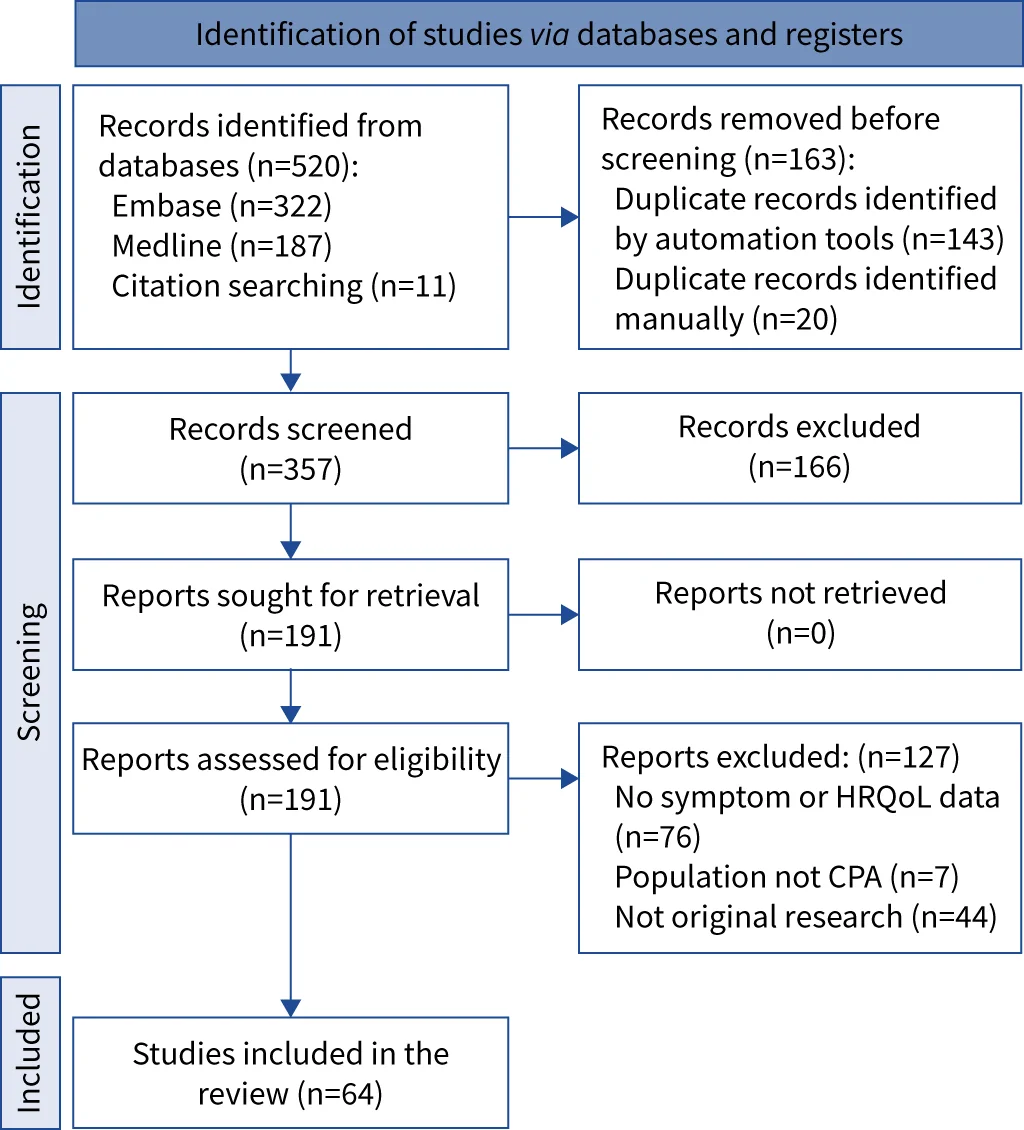
PRISMA (Preferred Reporting Items for Systematic Reviews and Meta-Analyses) flow diagram illustrating the study selection process. CPA: chronic pulmonary aspergillosis; HRQoL: health-related quality of life.

Most investigations were single-site observational studies that evaluated changes in HRQoL over time, capturing data at intervals including baseline, 3, 6 and 12 months, with nine studies specifically focused on investigating the impact of different therapeutic interventions on HRQoL. There was one randomised controlled trial (single-centre) with longitudinal HRQoL measurement at baseline and 3-monthly follow-up over 2 years [23].

HRQoL was predominantly assessed using generic HRQoL instruments, most commonly the St George's Respiratory Questionnaire (SGRQ) (n=16) and the Short Form 36 Health Survey Questionnaire (SF-36) (n=1). Several additional studies analysed symptom scores such as the Respiratory Symptom Score (RSS) (also identified as the 6 item-visual analogical scale) (n=1), the Visual Analogical Scale (VAS) for specific symptoms (*e.g.* cough, dyspnoea, haemoptysis) (n=4), the Medical Research Council (MRC) dyspnoea score (n=5), the Modified Medical Research Council dyspnoea score (mMRC) (n=1) and the Manchester COPD Fatigue Scale (MCFS) (n=1) (supplementary table S2) [2, 15, 16, 24–38]. Only one study formally validated the psychometric properties of the SGRQ in CPA [37], whereas subsequent studies primarily employed the SGRQ as an outcome measure for therapeutic response to antifungal treatments [16, 32, 34].

46 non-QBS reporting CPA-specific symptoms were published between 2009 and 2024 and drawn from geographically diverse settings. Most studies originated from India (n=12) and multi-country reports (n=9), with further contributions from the UK (n=5) and South Korea (n=5). Additional studies were conducted in Japan (n=4) and China (n=3), while single studies were reported from France, Morocco, the USA, Taiwan, Vietnam, Australia, Ghana and Poland (each n=1) (supplementary table S3) [3, 19, 27, 28, 39–81].

### CPA-specific symptom burden

To understand CPA-specific symptom burden, we reviewed the 46 included non-QBS analysing symptoms (figure 2). Haemoptysis was the most frequently documented symptom, reported in 38 studies (82.6%), followed by cough in 36 studies (78.3%) and dyspnoea in 34 studies (73.9%). Other common symptoms included weight loss (54.3%), fever (52.2%) and fatigue (50.0%). In contrast, nocturnal awakening (2.2%) and loss of appetite (4.3%) were rarely mentioned. Weight gain was identified as an indicator of favourable treatment response, with weight loss commonly linked to disease severity and worse QoL. This distribution highlights that CPA symptomatology is dominated by respiratory and constitutional features, with systemic manifestations being less consistently captured across studies.

**FIGURE 2:**
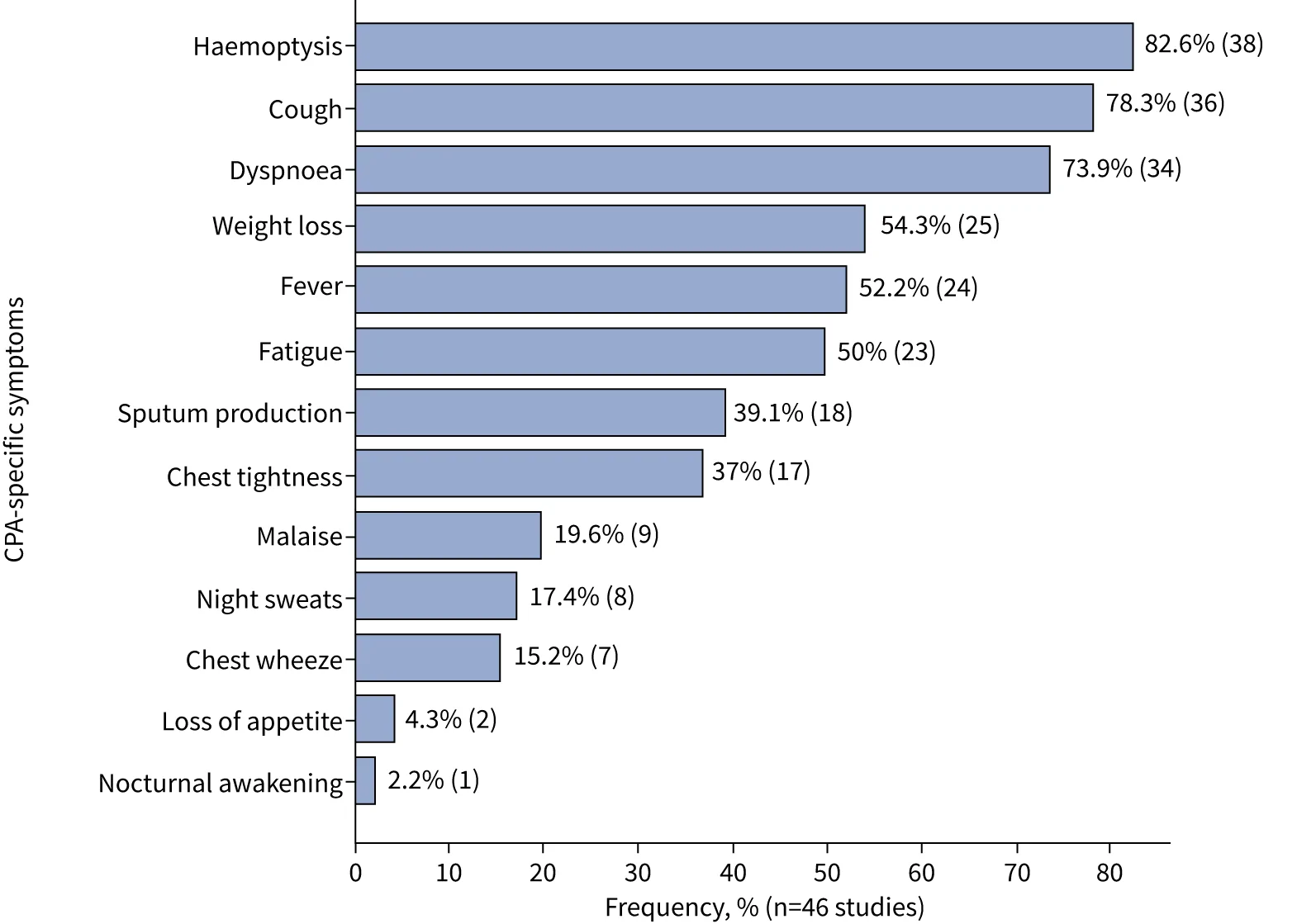
Most commonly reported chronic pulmonary aspergillosis (CPA)-related symptoms across included nonquestionnaire-based studies.

### Currently used HRQoL tools and symptom scores in CPA

Supplementary table S5 provides detailed descriptions of the purpose, target population, administration, structure and scoring of each HRQoL tool and symptom score in CPA (SGRQ, SF-36, RSS, MCFS, MRC, mMRC) as background information. In brief, the SGRQ and SF-36 are generic tools designed to measure health status in patients with chronic airflow limitations such as COPD and chronic conditions respectively. The RSS focuses on six cardinal symptoms, including cough, sputum, dyspnoea, haemoptysis, chest pain and nocturnal awakening, with the VAS providing a single-index measure of HRQoL or symptom severity. The MCFS is specifically designed to assess fatigue in respiratory diseases with the MRC dyspnoea scale evaluating breathlessness during daily activities within respiratory disease [82–84].

### Psychometric validity analysis: content validity

Table 1 summarises the psychometric validity of commonly used HRQoL tools in CPA. Given their generic nature, the content validity of currently used QoL tools in CPA have not been formally assessed (*e.g.* SGRQ, SF-36, VAS, MRC/mMRC and MCFS). Item generation was also developed without disease-specific qualitative study input including patient involvement and/or expert evaluation. As these tools are often derived to focus on respiratory (SGRQ, MRC) or fatigue (MCFS) domains, there are significant content omissions when analysing symptoms prevalent in CPA, particularly nonrespiratory items such as haemoptysis, fever and weight loss (figure 3). Similarly, the SF-36 only covers fatigue (*via* the vitality domain), which is captured (though indirectly). Even within respiratory domains, evidence from recent qualitative validation studies suggest inconsistency in patient interpretation of the mMRC dyspnoea scale as an example, emphasising the need for even widely adopted instruments to undergo disease-specific psychometric validation [85]. Prior studies have, however, supported the impact of fatigue as a component of HRQoL in CPA, with linear multivariate analysis showing fatigue as the strongest factor (beta=0.7; p<0.0001) associated with impaired health status determined through patient self-assessment grading, after adjusting for age, body mass index, forced expiratory volume in 1 s and MRC dyspnoea score [36].

**TABLE 1:** Comparison of the psychometric properties of existing health-related quality of life (HRQoL) tools and symptom scores in chronic pulmonary aspergillosis (CPA)

Property	SGRQ	SF-36	RSS	MCFS	MRC	mMRC
**Content validity and relevance to CPA symptoms**	Captures broad symptoms but lacks specificity; not derived from CPA patients/experts (n=46 non-QBS studies informing gap analysis) (figure 3)	Not derived from CPA patients/experts; captures fatigue only (vitality domain)	Items developed by consensus among 29 experts and two patients (n=1 study [26, 39]); respiratory symptoms only, omits constitutional symptoms	Measures fatigue only (n=1 study [35])	Assesses dyspnoea severity only (n=1 study [36])	Assesses dyspnoea severity only; qualitative inconsistency reported (n=1 study [19])
**Internal consistency**	Cronbach's alpha: total 0.94, symptoms 0.77, activity 0.91, impact 0.86 (n=1 study, N=88 [36])	NA	NA	Cronbach's alpha >0.98 (total), >0.93 (domains) (n=1 study, N=151 [35])	NA	NA
**Test–retest reliability**	ICC: total 0.97, symptoms 0.92, activity 0.94, impact 0.95 (n=1 study, N=17 [36])	NA	NA	NA	NA	NA
**Sensitivity**	>8-unit change: 90.9% sensitivity, 87.0% specificity for recurrence at 12 months (AUC 0.7); poor at 3 months (AUC 0.6) (n=1 study, N=77 [29])	NA	Partial: CPAnet definitions 59.1% sensitive for treatment failure *versus* 63.6% existing composite (n=1 study, N=43 [26])	NA	NA	NA
**Floor/ceiling effect**	NA	NA	NA	NA	NA	NA
**Responsiveness/MCID**	Total score MCID 7.3; domains: symptoms 4.5, activity 20.1, impacts 13.3 (anchor-based, VAS anchor; n=1 study, N=148 [15])	NA	Treatment response demonstrated (kappa=0.73 agreement with existing composite definitions); no formal MCID derived (n=1 study, N=43 [26, 39])	NA	NA	NA
**Convergent validity**	Correlated with MRC dyspnoea scale (r=0.51), VAS (r^2^=0.33), SF-36 (r=0.7) [15, 36]	Correlated with SGRQ (r=0.7) [36]	Agreement within CPAnet expert consensus; improved when weight loss added (kappa=0.73) [26, 39]	Correlated with SGRQ (r=0.83) (n=1 study, N=151 [35])	Correlated with SGRQ (r=0.51) [36]	NA
**Usability**	Not formally assessed (complex, three domains/50 items)	Not formally assessed	Not formally assessed	Not formally assessed	Not formally assessed	Not formally assessed

AUC: area under the curve; CPAnet: Chronic Pulmonary Aspergillosis Network; ICC: intra-class correlation coefficient; MCFS: Manchester COPD Fatigue Scale; MCID: minimal clinically important difference; mMRC: Modified Medical Research Council Dyspnoea Scale; MRC: Medical Research Council Dyspnoea Scale; NA: not assessed; QBS: questionnaire-based study; SF-36: Short Form 36 Health Survey Questionnaire; SGRQ: St. George's Respiratory Questionnaire; RSS: Respiratory Symptom Score; VAS: visual analogue scale.

**FIGURE 3:**
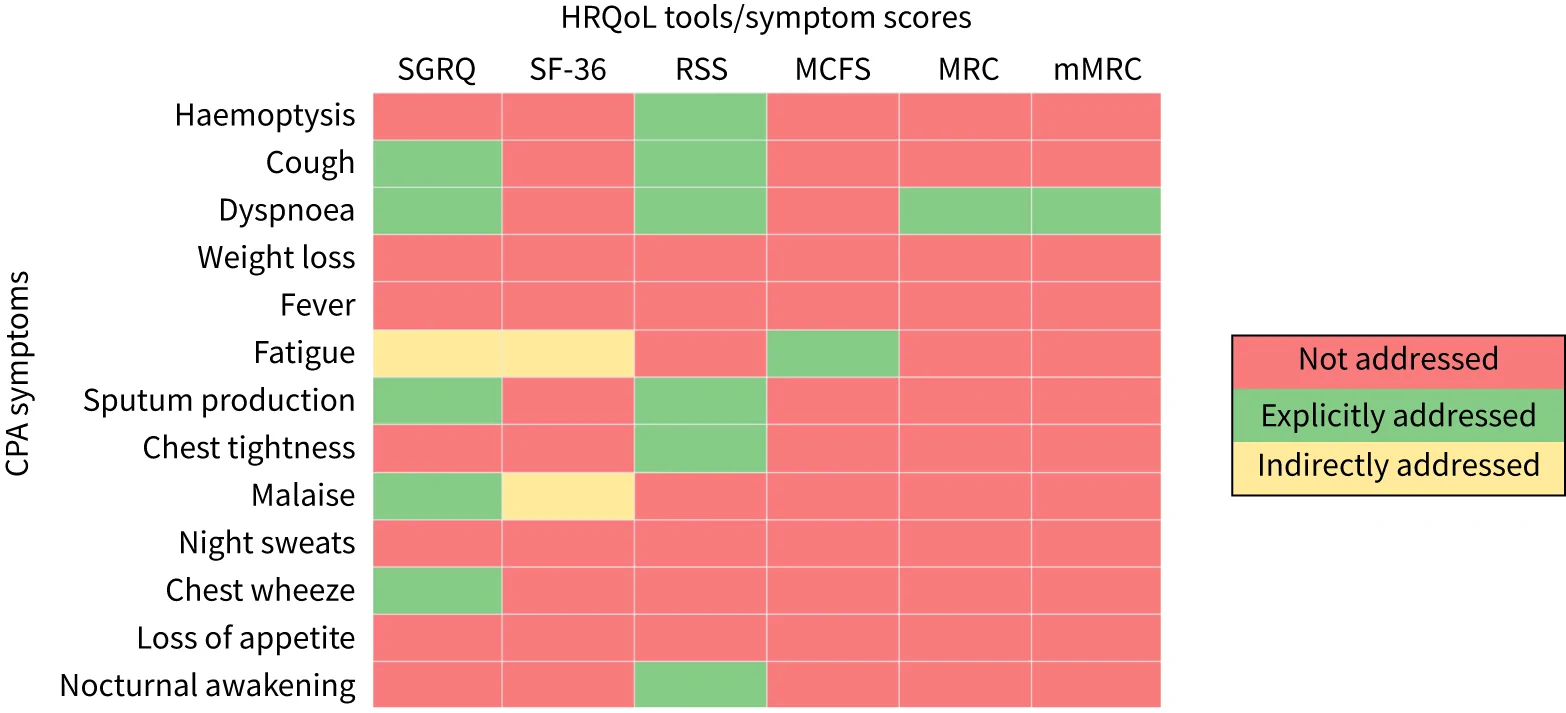
Symptom coverage mapping of chronic pulmonary aspergillosis (CPA)-relevant domains across health-related quality of life (HRQoL) tools and symptom scores used in the included questionnaire-based studies. Rows represent CPA-relevant symptoms and columns represent the instruments (St George's Respiratory Questionnaire (SGRQ), Short Form 36 Health Survey Questionnaire (SF-36), Respiratory Symptom Score (RSS), Manchester COPD Fatigue Scale (MCFS), Medical Research Council dyspnoea score (MRC), modified Medical Research Council dyspnoea score (mMRC)). Cells indicate whether each symptom is explicitly assessed (green), indirectly captured (yellow), or not assessed (red).

The RSS was the only symptom score that had partially established content validity in CPA based on consensus agreement among 26 experts out of 29 (96%) within the Chronic Pulmonary Aspergillosis Network (CPAnet) consortium [19]. The scale consists of a single symptom domain using a 10-cm visual analogue scale containing six items but lacks holistic QoL domains/items. Derivation of the score, however, was not based on a formal item/domain development process. A study evaluating the content validity of the CPAnet criteria by analysing treatment responsiveness in CPA, suggested improvements in content, including weight assessment for more complete coverage, indicating content validity gaps [27].

### Internal consistency

No formal studies have been conducted to assess the internal consistency of the existing HRQoL tools used in CPA other than the MCFS and SGRQ. Only one study has formally demonstrated that SGRQ is a reliable and valid tool for assessing HRQoL in patients with CPA and shows excellent internal consistency (Cronbach's alpha: total score=0.94, symptoms=0.77, activity=0.91 and impact=0.86). However, the retrospective nature of the study limited data validity; with the authors reinforcing the need for prospective, multicentre studies to confirm findings and better define the utility of the SGRQ in CPA [37]. A strong correlation was also seen between the MCFS and SGRQ, highlighting the significant impact of fatigue on HRQoL in CPA patients with the MCFS showing high internal consistency (Cronbach's alpha >0.98 for total fatigue and >0.93 for individual domains) [36].

### Test–retest reliability

SGRQ is the only tool in the current CPA literature with demonstrated test–retest reliability, with no equivalent repeatability data identified for the other HRQoL instruments/symptom scores used in CPA. In a single study, 17 clinically stable patients completed the SGRQ on two occasions 2–3 weeks apart, demonstrating excellent repeatability with an intra-class correlation coefficient (ICC) of 0.97 for the total score and similarly high ICCs across domains (symptoms 0.92; activity 0.94; impacts 0.95). Mean between-visit differences were small and nonsignificant (total +0.5±6.1, p=0.7), supporting the stability of SGRQ scores over short intervals when clinical status is unchanged [37].

### Sensitivity and treatment responsiveness

The sensitivity of the SGRQ symptoms domain was assessed in one retrospective cohort study evaluating clinical recurrence in CPA and reported that an >8-unit change at 12 months yielded high sensitivity (90.9%) and specificity (87.0%) for discriminating recurrence status. Overall discrimination of the symptoms domain at 12 months was moderate (receiver operating characteristic area under the curve (AUC) 0.7, 95% CI 0.6–0.8), while performance at 3 months was poor and nonsignificant (AUC 0.6, p=0.294) [30]. Improvements in SGRQ scores have been further reported with various long-term antifungal treatments, leading to substantial improvements in HRQoL (∼47%-50%), with a mean reduction in total SGRQ score of around 14 points at both 6 and 12 months [38]. In contrast, other studies however have shown more modest changes or deterioration in HRQoL attributable to treatment-related adverse effects [30].

CPAnet criteria for assessing treatment outcomes in CPA recommend the RSS as the standard measure of clinical response in CPA. A single retrospective study has evaluated the treatment responsiveness of the RSS by incorporating the CPAnet consensus outcome definitions, which incorporate the RSS as the clinical component, and compared them with an existing composite response assessment based on clinical, radiological and microbiological criteria in treatment-naïve CPA treated with 6-month itraconazole [27]. The CPAnet and existing definitions classified 69.8% and 67.4% of patients as treatment success, with substantial agreement (κ=0.73), but both missed eight patients who subsequently deteriorated and required treatment re-initiation within 3 months, with relatively limited sensitivity for detecting treatment failure (59.1% CPAnet; 63.6% existing composite assessment) [19]. Adding ≥5% weight loss as an additional worsening criterion improved sensitivity by ∼36%, highlighting a clinically relevant content omission of respiratory-specific symptom scoring in analysing treatment responsiveness in CPA.

### Minimal clinically important difference

The MCID for the SGRQ varies across lung conditions, typically requiring a change of ≥4 units in individual domains or ≥7–8 units overall to be clinically meaningful. Only one study has estimated an anchor-based MCID for SGRQ in CPA using the VAS, a patient-reported assessment of global sense of well-being, as the external anchor in 148 treatment-naïve CPA patients treated with 6-month oral itraconazole [15]. At 6 months, the MCID for the SGRQ total score was 7.3 points, with domain-level MCID estimates of 4.5 (symptoms), 20.1 (activity) and 13.3 (impacts). Using their clinical response categorisation, SGRQ scores did not differ significantly between those with favourable and unfavourable responses, and the authors noted that previous CPA studies have used an arbitrary, nonvalidated SGRQ threshold (≥10–12) to define treatment response or recurrence risk [34, 38].

Other studies in CPA have relied on the VAS to assess overall well-being or a single symptom domain, such as cough and haemoptysis, which have shown to improve with antifungal treatment. However, no studies have performed formal derivation of the MCID of these tools prior to their adoption [16, 23, 46].

### Convergent validity

The SGRQ has demonstrated convergent validity, correlating with clinical measures such as the MRC dyspnoea scale (r=0.51), VAS (r^2^=0.33) and SF-36 (r=0.7), further establishing its utility in assessing HRQoL in CPA patients [15, 36]. Similarly, the MCFS was formally validated in one study, which showed it correlates well with SGRQ scores (r=0.83), supporting that fatigue is a significant component of HRQoL in CPA [36].

### Usability

None of the studies included in this review assessed usability and acceptability (time of administration, complexity, length and clarity of the questions) of the various HRQoL tools and symptom scores in the context of CPA. Specifically, to note, while the SGRQ is a validated tool in CPA, given its complexity (three domains (50 items)) its usability has not been formally evaluated [37].

## Discussion

CPA is a debilitating global disease often associated with prolonged requirement for antifungal therapy with significant effects on HRQoL. In this systematic review, we comprehensively analyse existing HRQoL tools and symptom scores utilised in CPA and assess their psychometric validity. We highlight a lack of disease-specific HRQoL tools currently used in CPA, with a variety of generic scores currently used, most commonly the SGRQ. Additional symptom scores are utilised, however, these are often limited to one subjective aspect or domain of HRQoL or symptoms and as such do not provide a holistic assessment. Only the SGRQ had evidence of current comprehensive psychometric validity for use in CPA, showing it can discriminate between cases and controls at 12 months; however, the authors emphasised that it should not be used alone as a marker for CPA progression due to suboptimal performance [30]. Also, as a generic, lengthy and complex tool, there are significant limitations regarding content validity and with minimal assessment of usability.

The SGRQ aims to measure the burden of respiratory disease on HRQoL and has been validated and used to monitor therapeutic efficacy in many chronic pulmonary conditions, including COPD, asthma, idiopathic pulmonary fibrosis, cystic fibrosis, tuberculosis and CPA [86–89]. Our review shows a number of studies have shown treatment responsiveness of SGRQ scores of QoL following antifungal therapy in CPA with further studies showing that SGRQ scores could discriminate clinical recurrence post discontinuation of treatment [30, 33]. As such, the SGRQ has proven helpful in CPA as a measure of HRQoL as it shows acceptable reliability, responsiveness to treatment and correlation with severity. Limitations however remain in content validity as it does not capture critical HRQoL domains such as haemoptysis, fever and weight loss [25, 60], and as such cannot be used in isolation to monitor disease prognosis [25, 28, 90]. A previous study has highlighted significant correlation between the SGRQ and the SF-36 and MRC dyspnoea scores in predicting clinical recurrence suggesting a role for shorter simpler symptom/QoL tools in CPA; however, studies were single-centre and statistically underpowered, so further validation is required.

As current HRQoL tools in CPA are generic, none were developed from CPA patient-derived symptoms. As such, a majority of these tools fail to comprehensively capture core symptoms specific to CPA, such as haemoptysis, fatigue, weight loss, fever and malaise, which are frequently cited in the literature and identified as prevalent in our systematic review. Incorporating patient and public involvement and engagement is essential to ensure any HRQoL tool reflects patient priorities and is user-friendly [9]. This lack of content validity of generic HRQoL tools highlights a need for a disease-specific tool that includes CPA-specific symptoms to better reflect the lived experiences of individuals with CPA. A recently published consensus statement has suggested using the six-point RSS as a clinical tool to analyse therapeutic effectiveness. This was derived from a study analysing the efficacy and safety of voriconazole on the HRQoL of CPA patients using the VAS to evaluate the most common symptoms associated with CPA defined subjectively within the study [16]. The RSS score, however, is designed based on expert consensus rather than patient-led item/domain identification [49]. Best-practice guidance specifies that content validity requires direct patient input (*e.g.* qualitative interviews and cognitive debriefing), alongside expert review and literature-based item generation [9]. Symptom-frequency data extracted from published studies, as used in our review to characterise content validity gaps (figures 2 and 3), is a useful proxy for identifying omissions in existing tools, but it is not a substitute for patient-derived conceptual frameworks. By this standard, none of the tools currently used in CPA, including the RSS, whose items were derived by expert consensus with only two patients involved [49], meet best-practice criteria for content validity. As such, limitations remain with regard to patient perspective, an absence of holistic QoL content, highlighted content validity gaps (*e.g.* weight loss/nutrition, fatigue), with limited psychometric validity. To date, whether this is a robust tool for analysing disease-specific HRQoL in CPA is not clear.

A further, related conceptual limitation concerns disease attribution. CPA frequently coexists with other chronic structural lung diseases including post-tuberculous lung disease, COPD, ABPA, nontuberculous mycobacteria infection, sarcoidosis, bronchiectasis and prior lung malignancy [3–5]. In this context, it is often not possible to determine, from the generic HRQoL tools reviewed here, whether a given symptom or HRQoL impairment is attributable to CPA itself or to the underlying structural lung disease. This is compounded by the fact that the generic tools currently used in CPA (SGRQ, MRC/mMRC, MCFS) were developed for, and validated against, these underlying conditions rather than CPA meaning their scores may partly reflect the burden of comorbid disease rather than CPA specifically. Although evidence of treatment-responsiveness of generic tools as shown in our review highlight utility in CPA, this attribution problem helps explain, and is closely linked to, the content validity gaps identified above (*e.g.* omission of haemoptysis, fever and weight loss, which are more specific to active CPA than comorbid airways disease). Disentangling CPA-attributable symptoms from those of comorbid respiratory disease for example, through study designs incorporating comparator groups with the same underlying condition but without CPA is therefore a critical requirement for the interpretation of existing HRQoL data and for design and assessment of validity of future CPA-specific instruments.

The only CPA-specific evidence quantifying the sensitivity of the SGRQ in diagnosing CPA recurrence suggests that a symptom-domain change threshold (>8 units) can show apparently favourable sensitivity/specificity at 12 months. However, the moderate AUC and the lack of discrimination at earlier time-points indicate time-dependent performance and limit its utility for early identification. Also, caution was advised in interpretation of results given the retrospective design and sample size, supporting the use of SGRQ alongside, rather than instead of, broader clinical, radiological, and microbiological assessments when diagnosing CPA recurrence [30].

CPA is a heterogeneous condition, frequently coexisting with other chronic lung diseases, with substantial variability in symptom burden and limited randomised trial evidence, partly given the difficulty in evaluating therapeutic response. HRQoL tools therefore have an increasingly important role in evaluating treatment effectiveness in CPA, making rigorous assessment of their psychometric properties essential [9]. In our systematic review, only the SGRQ and the MCFS demonstrated evidence of internal reliability and sensitivity to change, whereas the RSS has shown treatment responsiveness in CPA, suggesting they may still be promising candidates for a short, focused HRQoL tool, pending formal validation.

The only identified study estimating MCID in CPA reported that the SGRQ total score threshold is ∼7 at 6 months, compared with the commonly cited cut-offs for other lung conditions, which are four (COPD), seven (idiopathic pulmonary fibrosis) and eight (ABPA), underscoring disease-specific variation [15]. However, this should be reported as an anchor-based estimate rather than a prospectively validated MCID. The anchor was a retrospective patient-reported VAS for global well-being (rather than a pre-specified global rating of change) and the study design did not permit validation against external clinical end-points. In that study, SGRQ scores also did not clearly discriminate between favourable and unfavourable clinical responders, consistent with the limitation that SGRQ is a generic HRQoL tool and may not capture CPA-specific symptom burden or treatment-relevant change. Collectively, these findings support cautious interpretation of SGRQ change thresholds in CPA and strengthen the case for developing and validating a CPA-specific HRQoL tool.

A recently published randomised controlled trial after inclusion dates for our systematic review provides further contemporary context for SGRQ-based responsiveness assessment in CPA treatment trials). The VICTOR-CPA trial compared oral itraconazole and voriconazole in 116 treatment-naive CPA patients in India, reporting comparable favourable clinical/radiological response rates at 6 months (69% voriconazole *versus* 67% itraconazole; p=0.84), but significantly more treatment-related adverse events with voriconazole (55% *versus* 34%; p=0.025). This provides confirmatory, contextual evidence that antifungal treatment trials in CPA continue to rely on SGRQ-based and clinical/radiological outcome definitions, reinforcing the relevance of the psychometric limitations discussed above to ongoing and future CPA trials [90].

Acceptability and usability are also key to making any HRQoL tool effective in clinical and research settings. CPA has a global burden and is managed across highly variable healthcare settings. HRQoL tools, therefore, need not only psychometric validation but also assessment of real-world usability and acceptability across languages, literacy levels and resource contexts (*e.g.* completion burden, comprehension, missingness and feasibility), alongside cross-cultural adaptation to ensure international comparability. We highlight the lack of usability analysis for the currently used HRQoL tools and symptom scores in CPA. The SGRQ, despite its thorough evaluation of respiratory health, is often criticised for being too lengthy, which may impede routine use in clinical and research settings [91]. In contrast, the VAS offers a simpler, quicker assessment of symptom severity, making it more suitable for routine clinical use; however, simplicity may come at the cost of content comprehensiveness. Data on the real-world frequency of use of these tools in clinical settings are limited, but anecdotal evidence suggests that tools like the VAS are more readily adopted due to their simplicity. This reliance on ease of use may inadvertently favour fewer comprehensive tools, potentially reducing the quality of symptom assessment in CPA. As such, incorporating usability analysis into prospective evaluation of HRQoL instruments is essential to ensure that tools are not only scientifically robust but also practically applicable.

Our study has some limitations as the study search was limited to the English language only and CPA has a global prevalence. Furthermore, given the variability in the measures used and the correlation data, a meta-analysis of HRQoL correlations with CPA symptoms was not feasible. Nevertheless, the limitations of the HRQoL tools reviewed in this systematic review reflect broader challenges in CPA research. Inconsistent definitions of treatment response across studies evaluating tools and scores, coupled with variability in how symptoms correlate with disease severity complicate understanding of health-related burden in CPA and potential application of HRQoL tools. Without a disease-specific CPA-specific HRQoL tool, comparisons of HRQoL across studies are currently limited and clinical outcome evaluation remains problematic. Additionally, the lack of CPA-specific psychometric evaluation for widely used tools such as the VAS and MCFS restricts their effectiveness and application, particularly in the context of randomised clinical trials for novel therapies. This underscores the need for a validated CPA-specific HRQoL tool derived from patient and health professional experts comprehensive enough to accurately capture the disease's unique impact on patients.

Building on the points above, we propose the following roadmap for future CPA-specific HRQoL tool development: 1) qualitative patient interviews and focus groups across diverse CPA populations and care settings to generate a candidate item pool grounded in patients’ lived experience; 2) expert clinician review and cognitive debriefing of candidate items to refine wording and ensure clinical relevance, including explicit consideration of how to separate CPA-attributable symptoms from those of comorbid respiratory disease; 3) field-testing and formal psychometric validation, including internal consistency, test–retest reliability, construct/convergent validity, responsiveness and MCID derivation in a prospective, multicentre cohort; and 4) cross-cultural and linguistic adaptation, given the global distribution of CPA and the geographic diversity of the cohorts identified in this review.

In conclusion, our systematic review highlights the lack of disease-specific HRQoL tools in CPA, the variability in generic tools currently used to measure HRQoL in CPA and their limitations in psychometric validity. No currently available HRQoL tool fully addresses the CPA-specific symptom profile, a gap highlighted in the CPA literature [15, 16, 28, 30]. This highlights a clear need for a validated CPA-specific HRQoL tool that encompasses the full range of symptoms relevant to CPA, through a systematic design and evaluation, to aid clinical monitoring and standardise therapeutic end-points in clinical trials.

**Points for clinical practice and future research**

- There are major content validity gaps of existing HRQoL tools in CPA, with widely used generic tools (*e.g.* the SGRQ) either not capturing key CPA symptoms, such as haemoptysis, fever and weight loss, or assessing single domains and not providing a holistic assessment.
- SGRQ is a valid HRQoL tool in CPA, but is complex and should not be used alone for monitoring given gaps in content validity.
- There is a need for a disease-specific CPA HRQoL tool derived from the perspectives of individuals with CPA to improve holistic assessment of CPA, treatment response and as novel therapeutic end-points for clinical trials.

## Data Availability

No datasets were generated or analysed during the current study.

## Supplementary material

**Please note:** supplementary material is not edited by the Editorial Office, and is uploaded as it has been supplied by the author. Supplementary material ERR-0039-2026.SUPPLEMENT
