# Supplementary material for: Health-related quality of life measures in chronic pulmonary aspergillosis: a systematic review of psychometric validity
Source: Eur Respir Rev. 2026 Nov 4;35(182):260039. doi: 10.1183/16000617.0039-2026 (PMC13732414; doi:10.1183/16000617.0039-2026)
Supplement: Supplementary file 1 [file ERR-0039-2026.SUPPLEMENT.pdf]

Supplementary Materials

Supplementary Table 1. Search strategy used for literature search (From databases' inception to 02 May 2025) in Ovid MEDLINE and Embase

**Ovid MEDLINE(R) ALL <1946 to May 02, 2025>**		
#	**Search Strategy**	**Results**
1	(chronic adj2 pulmonary aspergillos*).mp. [mp=title, book title, abstract, original title, name of substance word, subject heading word, floating sub-heading word, keyword heading word, organism supplementary concept word, protocol supplementary concept word, rare disease supplementary concept word, unique identifier, synonyms, population supplementary concept word, anatomy supplementary concept word]	924
2	(Quality adj2 life).mp. [mp=title, book title, abstract, original title, name of substance word, subject heading word, floating sub-heading word, keyword heading word, organism supplementary concept word, protocol supplementary concept word, rare disease supplementary concept word, unique identifier, synonyms, population supplementary concept word, anatomy supplementary concept word]	533277
3	"Quality of Life"/	305458
4	Health Related Quality of life.mp.	68831
5	(HRQOL or QoL).mp. [mp=title, book title, abstract, original title, name of substance word, subject heading word, floating sub-heading word, keyword heading word, organism supplementary concept word, protocol supplementary concept word, rare disease supplementary concept word, unique identifier, synonyms, population supplementary concept word, anatomy supplementary concept word]	86434
6	(survey* or questionnaire* or scale* or score* or index* or tool* or instrument* or measure* or checklist*).mp. [mp=title, book title, abstract, original title, name of substance word, subject heading word, floating sub-heading word, keyword heading word, organism supplementary concept word, protocol supplementary concept word, rare disease supplementary concept word, unique identifier, synonyms, population supplementary concept word, anatomy supplementary concept word]	9359615
7	health impact assessment/ or "surveys and questionnaires"/ or health care surveys/ or patient reported outcome measures/ or health surveys/ or health status indicators/ or chronic disease indicators/ or patient health questionnaire/ or self report/	774632
8	2 or 3 or 4 or 5 or 6 or 7	9576980
9	1 and 8	195
10	limit 9 to English language	187

Supplementary Materials

Embase Classic + Embase <1947 to 2025 May 02>		
#	**Search Strategy**	**Results**
1	(chronic adj2 pulmonary aspergillos*).mp.	1368
2	(Quality adj2 life).mp.	930947
3	"Quality of Life"/	723299
4	Health Related Quality of life.mp.	98439
5	(HRQOL or QoL).mp.	149688
6	(survey* or questionnaire* or scale* or score* or index* or tool* or instrument* or measure* or checklist*).mp.	12664643
7	health status indicator/ or health survey/ or chronic disease indicator/	255273
8	exp patient-reported outcome/	74632
9	2 or 3 or 4 or 5 or 6 or 7 or 8	13046356
10	1 and 9	332
11	limit 10 to English language	322

Supplementary Materials

Supplementary Table 2. Characteristics of the Questionnaire-Based Studies (QBS) assessing health-related quality of life in CPA.

Author, Year, (Ref)	Country	N	Study aim	Study design	How HRQoL data was measured	HRQoL Tool	Symptom Score	HRQoL-Related findings
Sehgal, et al., 2025 **(25)**	India	94	• To compare FDG-PET/CT parameters in persons with CPA achieving treatment success or failure after 6 months of oral itraconazole therapy.	Prospective observational study	At baseline and 6 months.	SGRQ	VAS	• The VAS outcome showed a significant reduction in symptoms, with the median VAS score decreasing from 60 mm at baseline to 18 mm at 6 months, indicating an improvement in symptoms.
Sehgal, et al., 2024 **(26)**	India	232	• To assess the clinical significance of Aspergillus sensitisation (AS) in CPA subjects with or without AS.<br>• To explore the association between AS and airflow obstruction on spirometry.	Retrospective observational study	At baseline and 6 months.	SGRQ	VAS	• CPA-AS patients reported poorer QoL compared to those without AS.<br>• Higher SGRQ total scores were observed in CPA-AS patients, indicating poorer QoL.<br>• The presence of AS was associated with worse QoL and a higher risk of airflow obstruction and CPA relapse.

Supplementary Materials

Namusobya et al., 2023 **(27)**	Uganda	162	• To determine the impact of CPA co-infection on the HRQoL of Ugandans with pulmonary tuberculosis (PTB) and to compare HRQoL between those treated for PTB alone and those with PTB complicated by CPA.	Prospective observational study, single-site	At baseline and post-treatment.	SGRQ	X	• Patients with PTB only had significantly better improvements in HRQoL as measured by SGRQ scores compared to those with PTB and CPA co-infection. • CPA co-infection negatively impacts HRQoL, as evidenced by poorer SGRQ scores in symptoms, activity, impact, and total scores at follow-up.
Sehgal, et al., 2023 **(15)**	India	148	• To estimate the minimal important difference MID for the SGRQ scores in subjects with CPA treated with oral itraconazole for 6 months.	Retrospective study, single-site	At baseline and post-treatment.	SGRQ	X	• MID for the (SGRQ) in CPA is 7.3, indicating a clinically significant improvement in QoL. The SGRQ scores showed significant improvement after 6 months of itraconazole treatment, with a median change in total score from 46.3 at baseline to 16.7 at 6 months.
Sehgal, et	India	43	• To evaluate the	Prospective	At baseline	X	VAS	• A substantial

al., 2023 **(28)**			agreement between existing treatment response definitions and the newly proposed CPAnet criteria for CPA.	observational study	and six months		RSS	agreement between both criteria but indicates limitations in sensitivity, and potential improvements by including weight change.
Sehgal, et al., 2022 **(24)**	India	164	• To compare the effect of 6-months of oral itraconazole with 12-months of oral itraconazole on CPA clinical outcomes, and to assess changes in quality of life and symptoms	Randomised controlled trial, Single-centre	At baseline, follow up for 2 years at 3-monthly interval	X	VAS<br>mMRC	• The 12-month treatment group showed greater improvement in VAS scores compared to the 6-month group, indicating better quality of life outcomes with longer treatment.
Ocansey et al., 2022 **(81)**	Ghana	41	• To determine the incidence of CPA at the end of pulmonary uberculosis (PTB) treatment and post-treatment, using Aspergillus-specific antibody testing as a screening tool.	Prospective observational study	At baseline, end of PTB treatment (T1), and 6 months post-treatment (T2)).	SGRQ	X	• The QoL outcomes, showed significant improvement for patients without CPA or treatment failure, with scores improving from an average of 51.4 to 3.8 at T1.<br>• In contrast, CPA patients had less improvement or worsening scores, with one patient's score decreasing

								from 45 to 29 at T1 and two new CPA patients' scores increasing from 14.5 to 36 at T2.<br>• Overall, patients without CPA had better QoL outcomes compared to those with CPA.
Bonogomin et al., 2021 **(31)**	UK	77	• To evaluate the utility of the SGRQ in predicting the likelihood of clinical recurrence of CPA in patients who come off antifungal therapy.	Retrospective clinical audit, single-centre, observational	Longitudinal	SGRQ	MRC dyspnoea scale	• Deterioration of more than 8 units in the symptom component of SGRQ was associated with clinical recurrence of CPA in patients who discontinued antifungal therapy.

Supplementary Materials

Bongomin et al., 2020 **(32)**	UK	49	• To evaluate the impact of quantitative baseline Aspergillus-specific IgG serum levels on weight changes and QoL outcomes of patients with CPA under antifungal treatment.	Retrospective observational study, single-site	At baseline, 6 months, and 12 months.	SGRQ	X	• Patients with high baseline Aspergillus-specific IgG levels were more likely to gain weight during antifungal treatment.<br>• There was no statistically significant difference in QoL outcomes between the groups at 6 and 12 months. Although Group A showed a trend towards better QoL outcomes at 6 months, it was not statistically significant.<br>• Overall, no association was found between baseline Aspergillus IgG levels and QoL outcomes.
Otu et al., 2019 **(33)**	UK	18	• To assess the safety and efficacy of intravenous micafungin administration for the treatment of CPA.	Retrospective observational study	At baseline and 3 months post-treatment.	SGRQ	X	• The clinical response to micafungin was lower than previously reported studies, with only a third of patients showing significant

Supplementary Materials

								improvement in QoL.
Bongomin et al., 2018 **(34)**	UK	206	• To assess the 'real life' impact of oral triazole therapy on QoL, body weight and serology over 12 months, specifically comparing itraconazole and voriconazole.	Retrospective observational study single-site	At baseline, 6 months, and 12 months.	SGRQ	MRC dyspnoea scale	• Antifungal treatment with itraconazole or voriconazole for at least 12 months improves QoL in CPA patients. • The mean improvement in SGRQ scores was 8.4 units for itraconazole and 9.3 units for voriconazole, indicating modest efficacy of azole treatment in improving QoL for CPA patients.
Rodriguez et al., 2018 **(35)**	UK	78	• To assess whether pre-set targets of improvement in SGRQ and weight gain can be used as criteria of response to posaconazole therapy in CPA.	Retrospective, non-controlled, observational, single-site	At baseline and at 4- and 6-months during treatment only.	SGRQ	X	• 44% of patients reported improvements in QoL, including improved appetite, enhanced energy levels, improved pulmonary symptoms, and better management of day-to-day tasks, as assessed by SGRQ.
Lowes et al., 2017 **(2)**	UK	387	• To assess the impact of various	Retrospective observational	At baseline only	SGRQ	MRC dyspnoea	• The SGRQ's activity domain was a

			objective and subjective factors on the mortality of CPA patients and to evaluate these factors as tools for assessing CPA prognosis.	Study, single-site			scale	significant predictor of survival, with higher scores indicating worse QoL and increased mortality risk.
Newton et al., 2016 **(36)**	UK	71	• To assess the clinical response and renal toxicity observed in CPA patients receiving short-courses of liposomal amphotericin B (LAmB) therapy.	Retrospective observational study, single-site	Pre-and post-treatment	SGRQ	RRS [VAS (0–100 mm)] . MRC dyspnoea scale	• QoL improvements were observed in 92.5% of patients receiving LAmB therapy, with significant improvements in SGRQ scores across symptoms, impact, activity, and total scores.<br>• A major improvement of 12 points was seen in at least 30% of patients. For repeated courses, improvements were noted in 91.7% of the courses.
Al-Shair et al., 2016 **(37)**	UK	151	• To investigate the validity and reliability of the Manchester COPD	Observational study, single-site	At baseline only	SGRQ	MCFS / MRC dyspnoea scale	• Fatigue significantly impacts QoL in CPA patients, as indicated by high SGRQ scores

Supplementary Materials

			Fatigue Scale (MCFS) in examining fatigue in CPA patients, study factors associated with fatigue and quantify its impact on health status.					and strong correlations between MCFS and SGRQ scores. · The Manchester COPD Fatigue Scale (MCFS) effectively captures the characteristics of fatigue in CPA, explaining 78.4% of its variance.

Supplementary Materials

Al-Shair et al.,2013 **(38)**	UK	88	• To examine the reliability and validity of the SGRQ in assessing health status in patients with CPA.	Observational study, single-site	Every 3 months)	SGRQ / SF-36	MRC dyspnoea scale	• The SGRQ demonstrated significant reliability and validity in assessing health status in CPA patients. It showed strong correlations with other health status measures like the MRC dyspnea scale and SF-36 components, effectively differentiating between disease severity levels and grades of shortness of breath.<br>• A four-point change in SGRQ scores is considered clinically significant.
Al-Shair et al., 2013 **(39)**	UK	122	• To assess the health status of patients with CPA and investigate the effect of long-term antifungal treatment on improving or stabilizing health	Prospective, observational study, single-site	At baseline and every 3 months over a 12-month period.	SGRQ	X	• Antifungal therapy improved health status and prevented CPA progression in most patients.<br>• 7%-50% of patients showed substantial improvement in health status, while

Supplementary Materials

			status.					32% deteriorated.<br>• Drug side effects were common and negatively impacted health gains.
Cadranel et al., 2012 **(16)**	France	48	• To evaluate the efficacy and safety of voriconazole as a first-line therapy for CPA.	Prospective, multicenter, non-comparative trial	At 6 months and at the end of the study.	SGRQ	X	• A significant improvement in QoL was noted at 6 months and at the end of the study, demonstrating a positive impact on patients' health status.

SUPPLEMENTARY MATERIAL

Supplementary Table 3. Characteristics of the Non-Questionnaire-Based Studies (N-QBS) reported CPA-specific symptoms.

Author	Reference	Year	country
Kitasato et al.	83	2009	Japan
Kohno et al.	52	2010	Japan
Ohba et al.	72	2012	Japan
Agarwal et al.	51	2013	India
Kosmidis et al.	3	2015	United Kingdom
Benjelloun et al.	62	2015	Morocco
Denning et al.	65	2015	Multi-country
Shin et al.	50	2016	South Korea
Yao et al.	67	2016	China
Uzunhan et al.	57	2017	France
Sehgal et al.	74	2018	India
Kosmidis et al.	47	2019	Multi-country
Sehgal et al.	48	2019	India
Barac et al.	49	2019	Multi-country
Bongomin et al.	32	2020	Multi-country
Asaad et al.	43	2020	United Kingdom
Osborne et al.	44	2020	United Kingdom
Sugimoto et al.	46	2020	Japan
Laursen et al.	55	2020	Multi-country
Shin et al.	58	2020	South Korea
El-Baba et al.	71	2020	USA
Lee et al.	78	2020	Taiwan
Lee et al.	80	2020	South Korea
Sehgal et al.	42	2021	India
Bongomin et al.	66	2021	Multi-country
Nguyen et al.	70	2021	Vietnam
Zarif et al.	75	2021	United Kingdom
Im et al.	76	2021	South Korea
Denning D.	77	2021	United Kingdom
Lee et al.	79	2021	South Korea
Braeckel et al.	19	2022	Multi-country
Sehgal et al.	24	2022	India
Zhong et al.	54	2022	China
Despois et al.	69	2022	Australia
Ocansey et al.	81	2022	Ghana
Sehgal et al.	28	2023	India

SUPPLEMENTARY MATERIAL

Sehgal et al.	29	2023	India
Agarwal et al.	40	2023	India
Piwkowski et al.	53	2023	Poland
Wang et al.	56	2023	China
Singhal et al.	59	2023	India
Evans et al.	60	2023	Multi-country
Barac et al.	61	2023	Multi-country
Jha et al.	63	2023	India
Soundappan et al.	64	2024	India
Palanivel et al.	68	2024	India

SUPPLEMENTARY MATERIAL

Supplementary Table 4. SWiM reporting checklist: Completed following Campbell M, McKenzie JE, Sowden A, et al. Synthesis without meta-analysis (SWiM) in systematic reviews: reporting guideline. BMJ 2020;368:l6890, as pre-specified in our PROSPERO protocol (Section 28).

Item	SWiM item description	How addressed in this review	Location in manuscript
1a	Provide a description of, and rationale for, the groups used in the synthesis (e.g., groupings of populations, interventions, outcomes, study design).	Studies were categorised into two groups: questionnaire-based studies (QBS), using HRQoL questionnaires or symptom-score indices; and non-questionnaire-based studies (non-QBS), using objective clinical, immunological, radiological, or microbiological assessment methods. This grouping was used because it distinguishes studies that generate directly comparable psychometric data (QBS) from studies used only to evaluate content validity/symptom coverage (non-QBS).	Methods, "Articles Classification Strategy"; Results, "Study selection and description"
1b	Detail and provide rationale for any changes made subsequent to the protocol in the groups used in the synthesis.	No changes were made to the QBS/non-QBS grouping itself, which was pre-specified in the protocol (Section 28).	Methods, "Data Source and Search Strategies"; Other Information, "Registration and protocol"
2	Describe the standardised metric for each outcome and any transformation methods used, citing methodological guidance consulted.	Not directly applicable in the sense intended by SWiM (developed primarily for synthesising intervention effect estimates). This review synthesises reported psychometric properties (Cronbach's alpha, intraclass correlation coefficient, correlation coefficients, sensitivity/specificity, kappa) rather than a single standardised effect metric; each property is reported in its own original unit/statistic as extracted, tabulated by instrument and property rather than transformed to a common scale.	Results, "Analysis of content validity and psychometric properties..."; Table 2
3	Describe and justify the synthesis method(s) used when meta-analysis was not possible.	A structured narrative synthesis was used, tabulating psychometric properties by instrument (Table 2) and CPA-symptom coverage by instrument (Figure 3). Meta-analysis was not undertaken owing to substantial heterogeneity in	Methods, "Data Synthesis"

		instruments, populations, study designs, and outcome definitions across the 64 included studies, and because most psychometric properties are reported by only a single primary study per instrument.	
4	Provide the criteria used, with justification, to select particular studies/estimates for the main synthesis, where more than one option existed.	All studies meeting eligibility criteria were included in the synthesis regardless of study quality (no risk-of-bias-based exclusion), consistent with the protocol. Where a psychometric property for a given instrument was reported by only one eligible study (the case for most cells in Table 2), that study's estimate is reported directly; no competing estimates required prioritisation.	Methods, "Eligibility Criteria"; "Data Synthesis"
5	State the method(s) used to examine heterogeneity in reported effects.	Heterogeneity in measurement approaches, recall periods, symptom coverage, and scoring systems across instruments was examined narratively and mapped visually (Figure 3, symptom-coverage heat map by instrument). Formal statistical heterogeneity testing (e.g., I^2) was not applicable, as most properties are supported by a single study per instrument rather than multiple comparable effect estimates.	Results, "Content validity and relevance to CPA-specific symptoms"; Figure 3
6	Describe the methods used to assess certainty of the synthesis findings.	A formal certainty-of-evidence framework (e.g., GRADE) was not applied, consistent with the protocol's specification that no formal risk-of-bias assessment would be conducted, as the review's purpose was descriptive extraction of HRQoL measurement use and validity rather than comparison of treatment effects. Study-level limitations (single-centre or single-cohort origin, retrospective design, small sample size) are stated alongside each finding (e.g., "Only 1 study...", "n=1 study, N=17") to allow readers to calibrate certainty.	Methods, "Risk of bias" (protocol); Results (each psychometric-property subsection); Table 2
7	Describe the graphical and tabular methods used to present the effects, and the study characteristics used to order studies.	Findings are presented in Table 2 (psychometric properties by instrument, with contributing study, sample size, and reference), Supplementary Table 4 (tool characteristics), Figure 1 (PRISMA flow diagram), Figure 2 (symptom-frequency bar chart across non-QBS studies), and Figure 3 (symptom-	Results; Table 2; Figures 1-3; Supplementary Table 4

		coverage heat map across QBS instruments). Studies are referenced by citation number throughout the text and tables.	
8	For each outcome, describe the synthesised finding and certainty, in language consistent with the review question, indicating which studies contribute.	Each psychometric-property subsection (content validity, internal consistency, test-retest reliability, sensitivity/responsiveness, MCID, convergent validity, usability) states the synthesised finding, explicitly names the contributing study/studies and sample size, and flags where only one small or methodologically limited study contributes (calibrating certainty accordingly).	Results, all subsections under "Analysis of content validity and psychometric properties..."
9	Report the limitations of the synthesis methods and/or groupings used, and how these affect the conclusions that can be drawn.	Discussed limitations include: meta-analysis was not feasible given outcome and instrument heterogeneity; most psychometric properties rest on a single, often small, single-centre or retrospective study, limiting generalisability; the QBS/non-QBS grouping, although pre-specified, cannot by itself resolve the disease-attribution problem raised by CPA's frequent coexistence with other structural lung diseases; and the search was restricted to English-language publications, which may have excluded tools evaluated in non-English literature.	Discussion (final section, "Our study has some limitations...")

SUPPLEMENTARY MATERIAL

Supplementary Table 5 - **Key characteristics of the existing HRQoL and Symptoms Score tools used in CPA.**

	**SGRQ** (90)	**SF-36** (93)	**RSS** (28,41)	**MCFS** (37,94)	**MRC** (95)	**mMRC** (18,85)
**Purpose**	Measures health status and QoL in respiratory conditions.	Measures general health status across various conditions.	Evaluates clinical response in CPA. [also identified as 6 items- visual analogical scale]	Measures fatigue burden.	Assess disability due to breathlessnes s in daily life.	Adapted version of the original MRC but refined for ease of clinical and research use.
**Target Population**	Patients with chronic airflow limitations (e.g., COPD, asthma)	General population.	Patients with CPA	Primarily COPD	General population.	For use in chronic lung disease (especially COPD).
**Administration**	Interview-based or self-administered	Interview-based or self-administered	Self-administered	Self-administered	Interview-based or self-administered	Interview-based or self-administered
**Length: Domains (Number of items)**	3 Domains (50-items with 76 weighted response):<br><br>• Symptoms<br>• Activity<br>• Impact	8 Domains (36 items):<br>• Physical Functioning<br>• Role physical<br>• Bodily pain<br>• General health<br>• Vitality<br>• Social functioning<br>• Role emotional<br>• Mental health	Single Domain, six cardinal symptoms:<br>• Cough<br>• Dyspnoea<br>• Sputum<br>• Haemoptysis<br>• Chest pain<br>• Nocturnal awakening	3 Domains (27-items):<br><br>• Physical<br>• Psychosocial<br>• Cognitive	Single domain (1-item):<br><br>• Functional limitation due to dyspnoea.	Single domain (1-item):<br>• Functional limitation due to dyspnoea; slightly broader clarity on exertional thresholds (e.g., walking pace, slope).
**Time to Complete**	8-15 minutes	10-15 minutes	2-3 minutes	5-10 minutes	<1 minutes	<1 minute
**Summary Score**	Total score and domain-specific	Does not produce a single overall	Stability is defined by a score	The total score ranges from 0 to	Single score (1 to 5); Each	Single score (0 to 4); categories

SUPPLEMENTARY MATERIAL

	scores (0-100; lower scores indicate better health status).	score. 8 domains, each scored on a 0 to 100 scale (higher scores indicate better health status); and a physical and mental component summary score.	variation between −25 and +25%. Significant improvement, defined by a decrease in the RSS>25%, based on the 6 items (10-cm line)	54; a higher score signifies more fatigue	category has specific descriptions of breathlessnes s related to certain activities.	are broader and less detailed.
**Cognitive Demand and Usability**	High; burdensome for severe symptoms; requires scoring algorithm	Moderate; less disease-specific wording	• Low; short symptom-based questions, easy for repeated assessment	Moderate-high; requires sustained attention and 2-week recall	Low; simple but less standardised phrasing	Low; simple, concise wording
